# Understanding creative teaching in twenty-first century learning among Islamic education teachers during the COVID-19 pandemic

**DOI:** 10.3389/fpsyg.2022.920859

**Published:** 2022-07-28

**Authors:** Hafizhah Zulkifli, Ab Halim Tamuri, Nor Alniza Azman

**Affiliations:** ^1^Faculty of Education, Universiti Kebangsaan Malaysia, Bangi, Malaysia; ^2^Institute of Teacher Education, Bangi, Malaysia

**Keywords:** education, creative teaching, teacher, twenty-first century learning, COVID-19

## Abstract

Education during the COVID-19 pandemic required teachers to be creative because students might not be able to complete education in a normal way. However, Islamic education teachers seem to lack the skills and attitudes required for twenty-first century learning, including creative teaching. The purpose of this study is to explore if Islamic education teachers were able to teach creatively by responding to twenty-first century learning during the pandemic. A qualitative methodology was adopted using a case study design. The sample consisted of four Islamic education teachers from Putrajaya schools. Our findings illustrated that there were six themes that emerged as elements of creative teaching in twenty-first century learning. These were: student-centered learning, twenty-first century teaching, creative inspirations, creative strategies, creative activities, and alternative evaluation. This study found Islamic education teachers to be creative in teaching and it demonstrated the professional development of Islamic education teachers.

## Introduction

Education during the COVID-19 pandemic was a great challenge. It entailed transforming education, from traditional teaching and learning to online teaching and from twenty-first century learning to online learning. This transformation pushed teachers to create an attractive teaching and learning environment capable of academic excellence and creative students in twenty-first century education (Pazin et al., 2022). Education in the twenty-first century is a student-centered learning process that includes elements of communication, critical thinking, creativity, collaboration, and values (Malaysian Ministry of Education, 2017). Hence, teachers need to be ready to play a crucial role in teaching students to acquire the elements of twenty-first century teaching and learning.

Teachers need to be aware of the specific challenges to education during the pandemic such as internet speed, inadequate bandwidth for online classes and materials downloading, conducting online exams, lack of support from parents, the inability of teachers to provide clear information, no lab sessions, and issues relating to evaluation of student's memorization of Quran (Mahyoob, 2020; Ibrahim and Abdul Razak, 2021), and accordingly create a desirable teaching and learning environment to implement creative teaching in the classroom (Pazin et al., 2022). To overcome these challenges, teachers need to be flexible in their preparatory methods and help students to adjust to the demands of studying at home during the pandemic-triggered lockdown (Daniel, 2020). To maintain the quality of teaching, teachers need to be creative to ensure that the teaching and learning process encourages students to experience meaningful learning (Jasni et al., 2020). The successful provision of a creative environment in education is largely dependent on teachers (Bereczki and Karpati, 2018). Hence, teachers need to be creative in teaching for developing unique and meaningful knowledge in the context of learning (Huang et al., 2019). However, the implementation of creative teaching in schools is poor and teachers do not make creative teaching a priority (Hong et al., 2017; Huang et al., 2019).

Schooling during the pandemic exposed the challenges in creative teaching, particularly the teachers' readiness and mastery of twenty-first century teaching. Hanapi et al. (2020) illustrated that the general level of preparedness among Islamic education teachers in the Klang valley was high, while their level of skill and attitude toward twenty-first century learning was moderate. Islamic education teachers also face an additional challenge due to the lack of teachers' training using technology in the Islamic education subject (Hanapi et al., 2020). These teachers believe that online content decreases the idea of creativity in teaching, and therefore, they lack technological rigor and neglect the innovation of applications (Suhid et al., 2021).

Additionally, the acceptance of twenty-first century learning by students is at a moderate level that requires teachers to improve their skills in teaching (Ismail and Ismail, 2018). Teachers too face problems understanding the concept of twenty-first century skills, especially creative elements (Ismail and Ismail, 2018). In addition, research by Mohd Nawi et al. (2020) revealed that Islamic education teachers have a moderate-high level of the use of technology.

A review of current literature shows that there are quite a few quantitative studies on the subject, such as Deng et al. (2020), Anderson et al. (2021), Chen and Yuan (2021), and Liu (2022). However, there is minimal research attention on qualitative studies (Mokhlis, 2019; Ismail and Mohamed Kassem, 2022) regarding this theme. Despite the volume of research on twenty-first century teaching and learning, little is still known about how teachers applied creative teaching during the pandemic using twenty-first century teaching and learning. Some of the literature discusses teacher readiness in teaching and learning, but very few explore the process of creative teaching during the pandemic. This knowledge gap will be addressed in our research.

This study also serves to benefit Islamic education teachers and encourage them to become better at creative thinking and teaching and come out with new ideas of teaching methods. As a result, students might be more focused, pay more attention to learning, express freely, improve problem-solving, and adopt a fun way to learn.

The purpose of this research is to understand the process of creative teaching among Islamic education teachers in their adoption of twenty-first century learning and assessment methods during the COVID-19 pandemic.

## Literature review

Research on creativity is quite extensive. Likewise, research on innovation and entrepreneurship is vast and encompasses all disciplines. Studies on creativity, innovation, and entrepreneurship focus on the individual and organizational levels with an emphasis on process, outcomes, and context/environment (Dino, 2015). Creativity is derived from the Latin word *creare* meaning ‘to make’ or ‘to produce’ and is used in Latin to denote the original ‘divine’ creation of the world. It embodied presenting something novel and original, thus, closely related to originality, novelty, inspiration, genius, and individuality (Preminger and Brogan, 1993, p 455-456). Creativity can also be defined as “the interaction among aptitude, process, and environment by which an individual or group produces a perceptible product that is both novel and useful as defined within a social context” (Plucker et al., 2004, p. 90) as cited in Dino (2015).

In addition, creativity is “the development of ideas about products, practices, services or procedures that are (a) novel or original and (b) potentially useful” (Amabile, 1996; Zhou and Shalley, 2003; Shalley et al., 2004, p. 934). It can also be defined as the “publicly visible attributes of a product presented by an actor to a field” (Ford and Gioia, 2000, p 707). Thus, we can say that creativity is a process of developing new ideas resulting in innovation that can be implemented in a specific context, and exhibits potential for entrepreneurship which then captures the opportunity to use it further.

Dino (2015) further differentiates creativity, innovation, and entrepreneurship. Creativity focuses on the generation of new or novel ideas or associations between existing concepts. Innovation focuses on the implementation of these ideas or concepts in some specific context, to produce outcomes that are original, useful, appropriate, and actionable. And entrepreneurship focuses on the identification and capture of opportunities for useful and actionable outcomes in which a need could be satisfied, the value created or a solution found for an intractable problem. To reinforce linkages among creativity, innovation, and entrepreneurship, an example of the Innovation Quest program was implemented at the California Polytechnic State University and the University of Connecticut. The aim was to foster student creativity, innovation, and entrepreneurship with the mission of creating jobs and companies. This program taught students what it takes to develop sustainable solutions in response to societal needs or market problems and in turn, set up a company/business.

In the context of education, creativity is necessary for teaching and learning. Teachers need to be creative in teaching to get and retain students' attention, and for them to remain motivated in the classroom. Creative teaching can also make learning invigorating, meaningful, and realistic. It is defined as “the development and use of novel, original, or inventive teaching methods” (Educational Resources Information Center (ERIC), 2017). It is a process whereby teachers provide innovative curricula and adapt teaching strategies according to the needs of their students. The focus is on the characteristics of knowledge and the teaching environment to achieve learning goals (Reilly et al., 2011; Liu et al., 2022). According to Palaniappan (2009) and Jasni et al. (2020), creative teaching is closely related to a variety of techniques and methods that incorporate elements of creativity and involve effective interactions between teachers and students (Tan and Goh, 2007; Palaniappan, 2009). Effective interaction between teachers and students includes transferring meaningful knowledge creatively (Rinkevich, 2011), improving teaching in terms of flexibility (Sawyer, 2004), and transferring open and existing knowledge to motivate and inspire students to explore and innovate (Mariani and Ismail, 2015).

Sawyer (2011), on the other hand, assessed creative teaching based on the teachers' ability to use their imagination and employ engaging methods, and possess the value of originality and judgment. Imagination was defined as “a mental ability that can transcend spatial and temporal limitations to form images. The ability is based on the combination of an individual's experience. This mental ability integrates the perceptual ability to visualize dynamic process, such as processing, transformation, reorganization, and mental innovation. Imagination enables an individual to have new ideas on things that they have never experienced, where these ideas are reflected in an individual's work, life, and plans for the future” (Chen and Yuan, 2021). Being closely related to creativity, imagination has a positive effect on teachers' creative teaching (Chen and Yuan, 2021) and it contributes to innovation and idea generation.

On creativity, the most commonly referred to are the theories of Amabile and Torrance. Sternberg and Lubart (1995) and Amabile (1996) proposed that creativity is no longer based on an individual's personality or thinking pattern, but based on how the individual's creativity is influenced by the interaction with the environment. Amabile (1983) hypothesized that a creative environment will be formed when (1) learning is seen as important and fun to students and teachers, (2) students feel valued, loved, and respected, (3) students are active in the classroom, (4) students are proud and feel the classroom and school belong to them, (5) teachers act as advisors, coaches and sources of knowledge, (6) students feel they can discuss their problems openly, (7) learning strategies that encourage collaboration are in place, and (8) learning is relevant to student life. On the other hand, Torrance (1961) constructed an intelligence test to measure creative thinking. There are four elements of creativity according to Torrance such as eloquence, flexibility, authenticity, and explanation. Similarly, Cheng (2001) (cited in Chen and Yuan, 2021) proposed assessing creative teaching based on five dimensions namely creative teaching ideas, ability in creative teaching, divergent thinking abilities in teaching, motives in creative teaching, and creativity in teaching performance. Teachers can measure their creative thinking based either on Torrance's four elements, or Cheng's five dimensions. Additionally, Sawyer (2011) forwarded imagination as a measure of creative teaching along with the possession of the value of originality and judgment.

In this study, we used the Torrance Incubation Model of Teaching and Learning (TIM). This model, originally developed by E. Paul Torrance, provides a framework for the development of lessons in three stages: first, Heightening Anticipation, where the teacher quickly draws the children in with something engaging. The purpose of the first stage is to create the desire to know, to learn, or to discover; to arouse curiosity; to stimulate the imagination, and to give purpose and motivation (Torrance, 1993). Second, Deepening Expectations, which is the heart of the lesson: what is being taught, why is it being taught, and how is it presented. The purpose of the second stage is to go beyond the surface or warm-up and to look more deeply into the new information (Torrance, 1993). Third, Extending the Learning, in which students consider the larger impact of what they learned during the extending the learning step, where their learning develops or “incubates.” The purpose of the third stage is to genuinely encourage creative thinking beyond the learning environment for the new information or skills to be incorporated into daily lives (Torrance, 1993). TIM is designed to provide powerful learning that promotes the development of creativity by infusing a creativity skill or concept into each stage and setting the stage for incubation to occur beyond the lesson.

Previous research on creative teaching found that factors that drive students' creativity in the classroom were positive relationships between teachers and students, modeling creative behaviors, ensuring a balance of freedom and structure, understanding students' needs and learning styles, creating opportunities for collaboration, and effectively using resources (Davies et al., 2014). For teachers, Pazin et al. (2022) claimed that there were three groups of factors that influenced teachers' creative teaching, namely demographic factors, individual factors, and organizational factors.

Creative teaching can improve collaborative, critical thinking, creative thinking, and communication skills; for example, the research by Susetyarini et al. (2022) claimed that there was an increase in the first and second cycles through problem-based learning. Creative teaching can also trigger the effectiveness of conducive and meaningful student learning as well as stimulate students' creative thinking (Freund and Holling, 2008).

## Materials and methods

This study employed qualitative methods using a case study. The case study methodology was best suited for answering the research questions on how Islamic education teachers taught during the pandemic and what was the evaluation system that was adopted in the virtual classroom.

This research adopted convenience sampling. The sample for this study was four primary school teachers in Putrajaya teaching Islamic education. The teachers were selected from Putrajaya because teachers here taught entirely online, and they are willing to share their experiences and permitted us to observe them online. They were teaching 11 and 12 year-olds and had a teaching experience between 6 to 8 years.

Data were collected through semi-structured interviews with four primary school teachers. Once the process of interviewing and data collection was done, our researchers completed the transcript and returned the transcript to the respondents (our sample of primary school teachers) for review. After the transcript was reviewed, the respondents signed the interview confirmation form to confirm the interview information. Certified interview transcripts were analyzed using an index. The process of indexing the transcripts was done by labeling a separate code for each respondent. The interview data were categorized, subcategorized, and analyzed using the coding method to generate themes.

Data were analyzed using thematic analysis as suggested by Braun and Clarke (2006), namely (1) entering data, (2) building code, (3) finding themes, (4) reviewing themes, (5) defining and naming themes, and (6) making a report.

For credibility, this study used peer assessment and consistency using the Cohen Kappa analysis and audit trail.

## Findings

Of the themes that emerged from the analysis, five themes were categorized under objective one: to understand the process of creative teaching among Islamic education teachers in twenty-first century learning. The five themes comprised student-centered learning, twenty-first century teaching, creative inspiration, creative strategies, and creative activities. One theme was identified for objective two: to understand the assessment used by teachers during the COVID-19 pandemic. The theme was alternative evaluation.

Table 1 shows the overall themes:

**Table 1:** Tables for codes themes.

	**Codes**		**Themes**	**Examples**
	**Category: student centered**	**Abbreviation**		
1.	SC: Critical Sc: Creative Sc: Communication Sc: Participate	SC-CRIT SC-CRE SC-COM SC-PAR	Themes 1: Student-centered learning	“As far as I know, twenty-first century learning is a student-centered learning process through communication, critic... critical and creative thinking as well as application of pure and ethical values.” (I1DU20-24/SC-CRIT-CRE-COM). “I am using talking chips methods as a technique in my classroom. The students can communicate when they have their chips, if they don't have their chips so they can't talk. These methods can improve their communication and had a good values. It means that they have to be patients if they want to talk (I1DU26-29/SC-COM) For example I asked my students to think the cause and effect about the bad attitude. One topic about respecting our parents in Islamic education, I had asked my students about the consequences if they don't respect their parents. Then my students will discuss among them the answer (I4DU 33-37) “For me, teaching and learning twenty-first century learning must focus on students. Students must actively participate in the classroom.” (I3DU29/SC-PAR).
	Category: twenty-first		Theme 2:	“The approach I always use is problem-based learning. I always use the i-Think Map
	century teaching		twenty-first	technique in Islamic education teaching. I use i-Think Map because it is a graphical
	CT: Problem	CT-PBL	century	compilation technique that helps students think creatively and critically.”
	based learning		teaching	(I1DU40-42/CT-PBL)
	CT: Project	CT-PRBL		“Usually, I will use 'Rally Robin', 'Time-Pair-Share', 'Think-Pair-Share', and acting
	based learning			techniques in Islamic education classes. I chose these methods because uh... they uh... seem
				to fit the subject I want. Most activities in twenty-first century learning are student-centered learning. So, students are trained to be self-reliant in finding information, solving problems, finding ideas with the resources provided. In this way, uh... it can help students improve their creative and critical thinking skills.” (I2DU 45-48) “I prefer to use the advice and simulation techniques approach as well as storytelling techniques.” (I3DU34). “I always use project-based learning to teach morals. For example, in moral with parent subtopic, I told them to make a video and make an act of respect for their parents. Later they share the videos and upload them to Google Classroom.” (I3DU 40-43/CT-PRBL) “These kids love to do projects, so I asked them to do a project for example in prayer practical topic. And then they do that prayer practical project individually and upload it to Google Classroom” (I2DU49-50) “Since the students have exercise books, so I do it alternately. For example, this week we use the textbook for learning, and then next week we look at YouTube. So there's a time we online and there's a time we offline.” (I4DU42-44) “I do teach online and offline. For example, on Monday and Thursday, we are online, while Friday we are offline, ha like that. If offline I asked my student to look up the work I uploaded on Google Classroom and do them.” (I3DU45-47) “I still use the Rally Robin, Time-Pair-Share, and Think-Pair-Share methods but I applied them through online.” (I2DU53-54)
	Category: Creative inspiration		Theme 3: Creative	Subtheme 1: Eager to Search for Information “For me, it was hard only at the beginning. But after many times online, I personally want
	CI: Searching information	CI-SI	inspiration	to find an application that I can use to teach online. Sometimes I ask friends what they are using. For example, there's this science teacher, he used YouTube, Peekaboo, Dr. Binocs
	CI: Ask friend	CI-AF		Show, right. So I was curious about what it was and what it was like to use it for Islamic
	CI: Imagine	CI-I		Education.” (I1DU50-54/CI-AF)
				“So I searched myself, find on the internet such as YouTube, Didik TV, and more for me to use in class. I don't refer to the teaching guidebook anymore.” (I1DU55-56/CI-SI) “Then it's easy to use the guidebook but now, I'm the type where I have to imagine first before I do something. Next, I planned and searched on the internet for online news and put them into my lesson plan.” (I2DU60-62/CI-I) “Now it happened to be my hobby for my brain to think about how to teach. Then I used to be comfortable referring to the guidebook and follow exactly like what it says. Now, I need to think and find out how to teach online well.” (I4DU62-64) Subtheme 2: Get out of their Comfort Zone “I don't refer to teaching guidebook anymore.” (I1DU60) “I used to love the guidebook but now, I'm the type where I have to imagine first before I do something.” (I2DU64) “Then I used to be comfortable referring to the guidebook and follow exactly like what it says.” (I3DU47) Subtheme 3: Think outside the box “I imagined first how I would do the online talking chip method. If in the previous teaching, we could use coins as a chip. If online what type of tools? So I imagine if I use Wheels of Names as a chip would it be okay.” (I2DU66-68/CI-I) “There's once after a headache online teaching, I wonder what to do to make these students happy to learn. I think and think and next, I had the idea of incorporating some methods into one lesson.” For example, I had combined lecture methods with Kahoot technique (I3DU50-53) “I am always open to any teaching methods that are appropriate to Islamic Education.” Actually I had create a new methods such as using short stories and students need to ask a question from the short stories. The questions must be higher order thinking skills. Normally teacher always tell a story, now I changed that. Students read that stories and create higher order questions from the short stories. (I4DU65)
	Category: Creative strategies		Theme 4: creative	‘Firstly, we need to set the time. Because of course during this Malaysian Movement Control Order time I need to manage between family and teaching time. Thus, I set up a
	CS: Find ideas CS: Mind mapping	CS-FI CS-MM	strategies	time at night ‘after Isya’ prayer, to started looking for new ideas to teach.” (I1DU61-63/CS-FI)
				“I always look for ideas on my day off. For example, I don't have class on Wednesday, so that day I will take time to think of the appropriate methods for future teaching. I tend to forget so I made a note in my hp.” (I2DU66-68) “Making mind mapping is easy. You used to tell your students to do mind mapping. Now I myself do the mind mapping method to teach and provide activities involving it during the process, usually the kids always create mind mapping during history period.” (I3DU73-75/CS-MM) “I love finding ideas for teaching. I always thought about how to get the students to understand when studying online. Sometimes I teach based on my own ideas.” …. I find my ideas through imagination, searching internet and sometimes asking my colleague. (I4DU67-70)
	Category: Creative activities		Theme 5: creative	“There are various activities I have done during this pandemic teaching; I first held the memorization competition for the chosen surahs and uploading them to telegram while
	CA: Online CA: Situation CA: Solve problem	CA-O CA-S CA-SP	activities	directly giving scores there. Secondly, I ask students to tell the story of the prophet Muhammad when he received the revelation and uploading them to Google Classroom.” (I1DU81-84)
	CA: Games	CA-G		“While teaching online, I asked the students to present in Google Meet and I also did Quizizz. Since of course we need to create an activity or else the students will be bored. Thus, I used the tools too.” (I2DU87-88/CA-G) “The activity that I see students love to do is when I use Quizizz where they can answer them together. I also made a Wordwall for them. I also do activities in groups using Jamboard.” (I3DU74-75/CA-G) “For the fiqh subject, I usually created a situation, for example during Istinja learning. Hence, I tried to create an istinja situation in the desert, so students need to think about how to solve it. If we give them an exemplary situation within Malaysia they can't really get it since Malaysia have a lot of water.” (I3DU76-79) “I usually put questions like situations questions in Google PowerPoint. In the class, it's easy for all students to write there and turn in. I create a circumstance where they need to think about, like what if they trampled on camel feces and how to purify it” (I1DU87-89/CA-S) “I also do give a realistic situation. For example, your sister doesn't perform salat, so how do you tell your mom?” (I2DU90) “I also give questions to students and ask them to solve them. For example, you had to take your dad's money on the table to buy food. Is it a proper thing to do?” (I4DU77-78/CA-SP) “I prefer to use games because I teach kids year 1 to year 3 so I always have them playing games like crossword, puzzles, dice games, and bingo. Sometimes I also do group activities using Wheels of Names.” (I4DU80-83/CA-G) “Ha! Right? It's very fun and exciting. Students are up to wonder, “What are we going to do tomorrow, Ustazah? Ala, isn't it over? It's no fun that after this is over, Islamic education is slower.” Ha they're very, very... Even someone gave me the idea, “We're going to play this thing.” Sometimes Ustazah follows the trick of studying so you can't play.” (I4DU89-92)
	Category: Alternative evaluation		Theme 6: alternative	“The headmaster asked me to do an online assessment too, so I did Quizizz. I give the students time to answer and ask the parents to set time at 9. So before 9 p.m., the students
	AL-assessment	AL-A	evaluation	need to be ready in Google Meet” (I1DU92-93) “I did my own assessment as well as using live worksheet” (I1DU97/AL-A) “I followed the mid-year test that headmaster asked to use Classkick, so I apply it and I also apply Quizizz” (I2DU100) “I do implement Classkick and Blooket” (I4DU97) “I use project assessments like asking students to do individual projects such as respect for guests and putting them on Google Slide” (I3DU98)

*(I1DU20-24) stand for: I-respondent, 1-number of the respondent, DU-Discourse Unit, 20-24 is the continuous number in paragraph*.

### Theme 1: Student-centered learning

For the question on what the respondents understood about twenty-first century learning, the respondents said twenty-first century learning focused on student-centered learning including critical thinking, collaboration, communication, and values. Few excerpts from their interview:

“As far as I know, twenty-first century learning is a student-centered learning process through communication, critical and creative thinking as well as application of pure and ethical values.” (I1DU20-24/ SC-CRIT-CRE-COM). Respondent one elaborated that she used talking chip methods to increase communication skills among students. Respondent one mentioned. “I am using talking chips methods as a technique in my classroom. The students can communicate when they have their chips, if they don't have their chips, they can't talk. These methods can improve their communication and have good values. It means that they have to be patient if they want to talk.” (I1DU26-29/ SC-COM)

Respondent four elaborated that twenty-first century learning urged students to think critically. “I usually ask my students to think critically. For example, I asked my students to think about the cause and effect of the bad attitude. In one topic about respecting our parents in Islamic education, I asked my students about the consequences if they don't respect their parents. Then my students will discuss among them the answer.” (I4DU 33-37)

Respondent three added that twenty-first century learning required students to be active in class. He mentioned that “For me, twenty-first century teaching and learning must focus on students. Students must actively participate in the classroom.” (I3DU29/SC-PAR).

### Theme 2: Twenty-first century teaching

In response to the researchers' question on what the respondents understood about twenty-first century teaching, they shared the technology they used. They commonly used problem-based learning, Rally Robin, Think-Pair-Share, and Fan and Pick. The respondents also used numerous twenty-first century teaching methods to suit the needs of the students in the classroom. A few excerpts from the interviews:

“The approach I always use is problem-based learning. I always use the i-Think Map technique in Islamic education teaching. I use i-Think Map because it is a graphical compilation technique that helps students think creatively and critically.” (I1DU40-42/CT-PBL)

“Usually, I will use 'Rally Robin', 'Time-Pair-Share', 'Think-Pair-Share', and acting techniques in Islamic education classes. I chose these methods because they seem to fit the subject I want. Most activities in twenty-first century learning is student-centered learning. So, students are trained to be self-reliant in finding information, solving problems, and finding ideas with the resources provided. In this way, it can help students improve their creative and critical thinking skills.” (I2DU 45-48)

“I prefer to use the advice and simulation techniques approach as well as storytelling techniques.” (I3DU34). “I always use project-based learning to teach morals. For example, in morals with parent subtopic, I told them to make a video and make an act of respect for their parents. Later they shared the videos and uploaded them to Google Classroom.” (I3DU 40-43/CT-PRBL)

“These kids love to do projects, so I asked them to do a project for example in prayer practical topic. And then they do that prayer practical project individually and upload it to Google Classroom” (I2DU49-50)

Respondents four and three employed blended learning as follows:

“Since the students have exercise books, so I do it alternately. For example, this week we use the textbook for learning, and then next week we look at YouTube. So, there's a time we online and there's a time we offline.” (I4DU42-44)

“I do teach online and offline. For example, on Monday and Thursday, we are online, while Friday we are offline, like that. If offline I ask my students to look up the work I uploaded on Google Classroom and do them.” (I3DU45-47)

“I still use the Rally Robin, Time-Pair-Share, and Think-Pair-Share methods but I applied them online.” (I2DU53-54)

### Theme 3: Creative inspiration

The respondents accepted that they must have creative inspiration to create meaningful learning. It involved searching for new information, getting out of their comfort zone, and thinking outside the box.

#### Subtheme 1: Eager to search for information

Respondent one was eager to search for information by asking friends. Excerpts:

“For me, it was hard only at the beginning. But after many times online, I personally want to find an application that I can use to teach online. Sometimes I ask friends what they are using. For example, there's this science teacher, he used YouTube, Peekaboo, Dr. Binocs Show, right. So, I was curious about what it was and what it was like to use it for Islamic Education.” (I1DU50-54/CI-AF)

Respondents one, two, and four sought additional information. Excerpts:

“So, I search myself, find on the internet such as YouTube, Didik TV, and more for me to use in class. I don't refer to the teaching guidebook anymore.” (I1DU55-56/CI-SI)

“Then it's easy to use the guidebook but now, I'm the type where I have to imagine first before I do something. Next, I planned and searched on the internet for online news and put them into my lesson plan.” (I2DU60-62)

“Now it happened to be my hobby for my brain to think about how to teach. Then I used to be comfortable referring to the guidebook and follow exactly like what it says. Now, I need to think and find out how to teach well online.” (I4DU62-64)

#### Subtheme 2: Get out of the comfort zone

Respondents one, two, and three said that they had to get out of their comfort zone to be creative. Excerpts:

“I don't refer to the teaching guidebook anymore.” (I1DU60)

“I used to love the guidebook; but now, I'm the type where I have to imagine first before I do something.” (I2DU64)

“Then I used to be comfortable referring to the guidebook and follow exactly like what it says.” (I3DU47)

#### Subtheme 3: Think outside the box

Respondents two, three, and four, shared that they were comfortable to think outside the box. Respondent two used imagination for the first steps, combined methods and created new methods. Excerpts:

“I imagined first how I would do the online talking chip method. If in the previous teaching, we could use coins as a chip. If online what type of tools? So, I imagine, if I use Wheels of Names as a chip, would it be okay?” (I2DU66-68/CI-I)

“There's once after a headache online teaching, I wonder what to do to make these students happy to learn. I think and think and next, I had the idea of incorporating some methods into one lesson. For example, I had combined lecture methods with Kahoot technique.” (I3DU50-53)

“I am always open to any teaching method that is appropriate for Islamic Education. Actually, I had created a new method such as using short stories and students need to ask a question from the short stories. The questions must be higher-order thinking skills. Normally teachers always tell a story, now I change that and students read those stories and create higher-order questions from the short stories. (I4DU65)

### Theme 4: Creative strategies

Knowing that all the respondents employed creative teaching in the classroom, we explored what strategies they adopted to start creative teaching such as setting the time, free time, mind mapping, and finding ideas. Few excerpts:

“Firstly, we need to set the time. Because of course during this Malaysian Movement Control Order time I need to manage between family and teaching time. Thus, I set up a time at night after *Isya'* prayer, to start looking for new ideas to teach.” (I1DU61-63/CS-FI)

“I always look for ideas on my day off. For example, I don't have class on Wednesday, so that day I will take time to think of the appropriate methods for future teaching. I tend to forget so I made a note in my hp.” (I2DU66-68)

“Making mind mapping is easy. You used to tell your students to do mind mapping. Now I myself use the mind mapping method to teach and provide activities and involve it during the process. Usually, the kids always create mind mapping during history period.” (I3DU73-75/CS-MM)

“I love finding ideas for teaching. I always thought about how to get the students to understand when studying online. Sometimes I teach based on my own ideas. …. I find my ideas through imagination, searching the internet, and sometimes asking my colleagues.” (I4DU67-70)

### Theme 5: Creative activities

The pandemic gave the respondents an opportunity to implement creative activities such as quizzes, online *hafazan* (memorization) competition, hypothetical situations, real situations, and games. Few excerpts from what the respondents shared:

“There are various activities I have done during this pandemic teaching; I first held the memorization competition for the chosen *surahs* and uploaded them to Telegram while directly giving scores there. Secondly, I ask students to tell the story of the prophet Muhammad when he received the revelation and uploaded them to Google Classroom.” (I1DU81-84)

“While teaching online, I asked the students to present in Google Meet and I also did Quizizz. Since of course we need to create an activity or else the students will be bored. Thus, I used the tools too.” (I2DU87-88/CA-G)“The activity that I see students love to do is when I use Quizizz where they can answer them together. I also made a Wordwall for them. I also do activities in groups using Jamboard.” (I3DU74-75/CA-G)

“For the *fiqh* subject, I usually created a situation, for example during *Istinja* learning. Hence, I tried to create an *istinja* situation in the desert, so students need to think about how to solve it. If we give them an exemplary situation within Malaysia they can't really get it since Malaysia have a lot of water.” (I3DU76-79)

“I usually put questions like situations questions in Google PowerPoint. In the class, it's easy for all students to write there and turn it in. I create a circumstance where they need to think about, like what if they trampled on camel feces and how to purify it” (I1DU87-89/CA-S)

“I also do give a realistic situation. For example, your sister doesn't perform *salat*, so how do you tell your mom?” (I2DU90)

“I also give questions to students and ask them to solve them. For example, you had to take your dad's money on the table to buy food. Is it a proper thing to do?” (I4DU77-78/CA-SP)

“I prefer to use games because I teach kids year 1 to year 3 so I always have them playing games like crossword, puzzles, dice games, and bingo. Sometimes I also do group activities using Wheels of Names.” (I4DU80-83/CA-G)The respondents revealed that when they planned creative activities for their students, the students enjoyed and had fun learning Islamic education subjects.

“Ha! Right? It's very fun and exciting. Students are up to wonder, “What are we going to do tomorrow, *Ustazah*? *Ala*, isn't it over? It's no fun that after this is over, Islamic education is slower.” Even someone gave me the idea, “We're going to play this thing.” Sometimes *Ustazah* follows the trick of studying so you can't play.” (I4DU89-92)

### Theme 6: Alternative evaluation

After teaching, the respondents created assessments for the students to assess the students' understanding of the topic. During the pandemic, the respondents used alternative evaluations such as Classkick, Quizizz, Blooket, and a final project. The respondents explain the alternative evaluations they adopted in the following excerpts:

“The headmaster asked me to do an online assessment too, so I did Quizizz. I give the students time to answer and ask the parents to set time at 9. So before 9 p.m., the students need to be ready in Google Meet” (I1DU92-93)

“I did my own assessment as well as using live worksheet” (I1DU97/AL-A)

“I followed the mid-year test that headmaster asked to use Classkick, so I apply it and I also apply Quizizz” (I2DU100)

“I do implement Classkick and Blooket” (I4DU97)

“I use project assessments like asking students to do individual projects such as respect for guests and putting them on Google Slide” (I3DU98)

An assessment of our observation and document review showed that our researchers played a role as complete observer. The researchers were either hidden from the group or were in a completely public setting. Our findings from observation confirmed that the respondents implemented the twenty-first century learning to the students and added an element of creativity in their teaching. The respondents were very efficient in undertaking twenty-first century teaching and the students were active and had fun learning online. The respondents also included values in the teaching and learning such as reciting the ‘Doa’ (Prayer) before class and ending it with *tasbih kafarah*. Our document analyses also reconfirmed our observational findings regarding the teaching and learning process. The researchers investigated the lesson plan, students' scores from Quizizz or alternative evaluations—either formative or summative assessment documents, and project papers submitted by students as assignments.

## Discussion

Our study found a total of six themes in our attempt to understand creative teaching in twenty-first century learning among Islamic education teachers during the COVID-19 pandemic. These were student-centered learning, twenty-first century teaching, creative inspiration, creative strategies, creative activities, and alternative evaluation.

### Student-centered learning

All respondents believed that learning should be student-centered because it gave students freedom in learning. The concept of student-centered learning was to bring the classroom alive for the students. The teacher is considered a “guide on the side”, assisting and guiding students to meet the goals determined by the students and the teacher. Islamic education teachers need to master teaching in the student-centered mode, focusing on the most prominent twenty-first century skills such as critical thinking, creative thinking, collaboration, communication, and values (Overby, 2011; Ministry of Education, 2017). This is similar to the research by Jusof and Hamzah (2020) showing that Islamic education teachers need to maintain student-centeredness on three levels: student-centered knowledge, student-centered skills, and student-centered practices. The student-centered learning has been applied in the respondents' classrooms as they had been trained well at teacher education institutions.

### Twenty-first century teaching

There are various twenty-first century teaching methods such as problem-based learning, i-Think, and role-play. The respondents applied these twenty-first century teaching methods as they had been urged by their academic administrators or principals to make their teaching and learning meaningful. Research by Daud and Ab Rahman (2020) showed that the i-Think map was effective in teaching and easy to use. The i-Think map stimulated students to think and be more active during the teaching and learning process as well as become more creative and engaging (Awi and Zulkifli, 2021). Islamic education teachers should incorporate the i-Think map and similar technology and pedagogy skills to transform their curriculum with elements of creativity (Awi and Zulkifli, 2021). Subri et al. (2012) pointed out that there were positive improvements in teaching shariah when using one of the twenty-first century learning methods, such as role-playing. It resulted in the students becoming more creative while giving presentations, more comfortable, and enjoying themselves learning shariah.

### Creative inspiration

Teachers need to explore a variety of teaching methods to generate meaningful learning. They need to be creative to think differently and ensure that all students can learn and understand the topic. No child should be left behind. This is supported by Zolfaghari et al. (2011), who stressed that to manifest creativity, teachers need to be creative in teaching by asking creative questions, inspiring students' imagination, soliciting solutions, responding to open-ended questions, and finding similarities and differences. Hamed (2015) points out that the main factors that influence creative teaching are personal effort, teaching belief, teaching commitment, and personal knowledge.

### Creative strategies

Most respondents developed their strategy to be creative in teaching. For instance, some would jot down notes, set time, mind map, and come up with their own ideas in teaching. It is supported by Joubert (2001, p. 21) who observed that creative teaching is an art. One cannot teach teachers didactically how to be creative as there is no fail-safe recipe or routine. Some strategies may help to promote creative thinking, but teachers need to develop a full repertoire of skills that can be adapted to different situations. Creative strategies reflect the teachers' ethics in generating and exploring new ideas, encouraging autonomy and agency, playfulness, problem-solving, risk-taking, co-constructing and collaborating, and teacher creativity. This finding is in agreement with Wan Abdullah and Abdul Razak (2021) who summarized that to be a creative and innovative teacher, one should begin with the intention and desire, explore new knowledge, scribble down the issues, motivate self-interest, and seek new ideas.

### Creative activities in teaching

Islamic education teachers need to be creative in teaching by including creative activities in the classroom. Distinction needs to be made between creative teaching and teaching for creativity. While the former is focused on teacher orientation, the latter is related to learner orientation. Creative teaching requires teachers to make learning more interesting and effective by using imaginative approaches in the classroom. Teaching for creativity, in contrast, requires teachers to identify students' creative strengths and foster their creativity. Among the platforms that are often used when teaching online are WhatsApp, Google classroom, YouTube, and Google Meet. Other platforms including Gmail, Padlet, Edmodo, Quizizz, and Wordwall are rarely used. This also shows that they are comfortable using the platforms recommended by the Ministry of Education Malaysia (82.5%). Even so, 20 respondents found it less comfortable using online teaching (25%) and they preferred to give assignments to students manually either through mail or required students to come physically to the school to pick up the learning materials (43.75%) (Hashim et al., 2020). Our findings also showed that some respondents used Quizizz, Wordwall, hypothetical situations, real situations, Wheels of Names, and open-ended questions. The respondents explored creative activities to make sure the process of learning was fun and meaningful. This finding is supported by Lubis et al. (2017) who found that the level of knowledge of multimedia among Islamic education teachers in secondary schools in Selangor was quite high. Equally, the readiness of Islamic education teachers to use multimedia and related technology was moderate to high. When the knowledge and skills of Islamic education teachers are high, they can organize creative activities. This finding was also consistent with the study by Zulkifli et al. (2021) who demonstrated that the level of creative teaching practices among polytechnic Islamic Education lecturers in central and southern Malaysia is quite high.

### Alternative evaluation

Classroom assessment encompasses any form of behavior, interaction, and activities either planned or not, and explicit or integrated with the potential to transfer knowledge. This includes assessment of the development of students' achievement individually or in a group. Assessment in a classroom context is commonly associated with formative assessment (Webb and Jones, 2009). The teacher can ask students to create portfolios, do specific projects or engage in any other type of activity. During the COVID-19 pandemic, most respondents used Classkick and projects. The research by Chua et al. (2021) used a quasi-experiment to examine how Classkick influenced students' knowledge and the teacher's teaching practices. They found that Classkick enhanced the quality of learning and changed the way students learn. It helped teachers to customize their instructions based on individual student's needs.

## Conclusion

Creative teaching has become essential in today's classroom, especially after the COVID-19 pandemic. To address the damage caused by the pandemic and its lockdowns, and to avoid dropout among students, this study reveals that, Islamic education teachers in Putrajaya adopted twenty-first century teaching and successfully implemented it in their online classrooms. Creative teachers can think and come out with new ideas in teaching and learning to enhance the quality of teaching.

The are several limitations to this study. This study focused on creative teaching in twenty-first century learning. Thus, it does not fully capture the other elements of twenty-first century teaching, especially critical teaching, collaborative teaching, and values teaching. It is possible that future research can find different elements of twenty-first century learning in the future. Next, this study was limited to exploring the process of creative teaching only. Hence, it does not fully cover the characteristics, problems, and challenges in teaching during the pandemic. Future studies should include characteristics of creative teachers, how they help students to be creative, and the problems and challenges they face to see different results. Noting that this research is from the perspective of teachers in urban areas, future research could be undertaken in rural areas to assess if the results are different. This study used teachers as the sample. Other research could include students as samples. This research is a qualitative method employing a case study research design. Future research could assess creative teaching using action research. It is also suggested that different methods should be used, such as quantitative methods including survey, correlation, and experimental design to explore the factors that influence creative teaching among Islamic education teachers, examine their readiness in creative teaching, and evaluate their performance. This study is limited in its transferability as the number of participants who volunteered were few and from different public schools; hence the sample might not represent the whole population of Islamic Education teachers.

## Data availability statement

The raw data supporting the conclusions of this article will be made available by the authors, without undue reservation.

## Author contributions

HZ conceived and designed the study. NAA contributed to the literature review. HZ and AHT collected and organized the database, undertook the analysis, and discussion. All authors read, revised, and approved the final manuscript.

## Funding

This research was funded by Universiti Kebangsaan Malaysia, Grant Number GG-2020-030, and the APC was funded by the same grant.

## Conflict of interest

The authors declare that the research was conducted in the absence of any commercial or financial relationships that could be construed as a potential conflict of interest.

## Publisher's note

All claims expressed in this article are solely those of the authors and do not necessarily represent those of their affiliated organizations, or those of the publisher, the editors and the reviewers. Any product that may be evaluated in this article, or claim that may be made by its manufacturer, is not guaranteed or endorsed by the publisher.
